# The Wearable Co-Design Domino: A User-Centered Methodology to Co-Design and Co-Evaluate Wearables

**DOI:** 10.3390/s20102934

**Published:** 2020-05-21

**Authors:** Leire Francés Morcillo, Paz Morer-Camo, María Isabel Rodriguez Ferradas, Aitor Cazón Martín

**Affiliations:** Department of Mechanical Engineering—Industrial Design Area, University of Navarra, 20018 San Sebastian, Spain; pmorer@tecnun.es (P.M.-C.); mirodriguez@tecnun.es (M.I.R.F.); acazon@tecnun.es (A.C.M.)

**Keywords:** co-design, wearable, multidisciplinary, human-centered design, toolkit, wearable computer

## Abstract

This paper presents a user-centered methodology to co-design and co-evaluate wearables that has been developed following a research-through design methodology. It has been based on the principles of human–computer interaction and on an empirical case entitled “Design and Development of a Low-Cost Wearable Glove to Track Forces Exerted by Workers in Car Assembly Lines” published in Sensors. Insights from both studies have been used to develop the wearable co-design domino presented in this study. The methodology consists of different design stages composed of an ideation stage, digital service development and test stages, hardware development and test stage, and a final test stage. The main conclusions state that it is necessary to maintain a close relationship between human factors and technical factors when designing wearable. Additionally, through the several studies, it has been concluded that there is need of different field experts that should co-design and co-evaluate wearable iteratively and involving users from the beginning of the process.

## 1. Introduction

The research field of wearable has grown during the last decades. Recent years have seen increased interest in the design of digital and physical–digital hybrids due the digital revolution that has added new dimensions to the field of wearable research.

Although wearable boomed in the 1960s, wearable have been around for a long time and their role has been changing according to the historical and technological context of the moment. While wearables were perceived as miniature versions of existing products at first, they are huge service hubs nowadays. Thus, the creation of wearable requires specific concepts, techniques and ingredients involving textile, electronics and software that consider the diversity of potential users and their environments. This evolution of wearable can only be understood through the close inter-relation between new technological developments and people’s needs.

Even though this reality involves understanding wearable through the close inter-relation between technological developments and people needs, there are few studies that follow this research line. Overall, there is a lack of a dialogue between human and technical factors both in the design process and in the validation process. This means that, most of the information found in literature is focused on mainly technical or human factors but not on a shared focus. Having identified this research opportunity, this paper seeks to provide a combined view of both technical and humans factors across a user centered methodology. To achieve this and due to the wearable evolution, it is important to define how the “wearables” is going to be defined.

As there are multiple definitions of what a wearable device is, this study has taken the following as reference: “a wearable is a fully functional, self-powered, self-contained computer that is worn on the body, providing access to and interaction with information anywhere and at any time” [1]. This definition is not based on a particular view, but it’s a general definition that can be applied to different contexts. Moreover, both technical and human aspects are considered and the wearables main requirements mentioned above (wearability, computer technology, and connectivity) are considered. Due to these reasons, the author considered that this definition is appropriate to this paper.

Taking all the above issue into account, this paper is aimed to identify the role of user-centered design in the development of wearables. It consisted of four studies that have been attuned to answer specific research questions and that has led to a research-through-design method combining design and research activities.

Firstly, this paper will present the related theoretical background by reviewing the user-centered methodologies that can be applied to the development of wearables. From this analysis, key principles for different methodologies will be identified in order to develop the theoretical framework of the methodology.

Secondly, the different studies carried out will be represented. Some of these studies are explained into detail referring to different indexed publications [2] in a conference publication [3]. During the development of the paper, a causal relationship between different studies has been developed. This means that outcomes from the first study has guide the incomes for the following study and thereby, consecutively.

As a result of this process, the main contribution of this paper is the wearable co-design domino: a methodology to co-design and co-evaluate wearable. In this study, the methodology and the requirements will be first presented. Then, the methodology inspiration and implementation process, and then, the design proposal and the validation. Once the validation has been explained, the full methodology description will be explained. 

Finally, a discussion and conclusions are presented in the paper.

## 2. Review of the Literature

### 2.1. Wearables’ Evolution

As it has been mentioned before, wearables date from a long time ago and their meaning and has been changing along the years. To better understand the evolution and the current wearables’ situation, this section describes the evolution of wearables based on the different technological phases that have been identified along the time. This focus, helps to understand wearables current perception and why a multidisciplinary approach is necessary to design and develop them.

**Figure 1:**
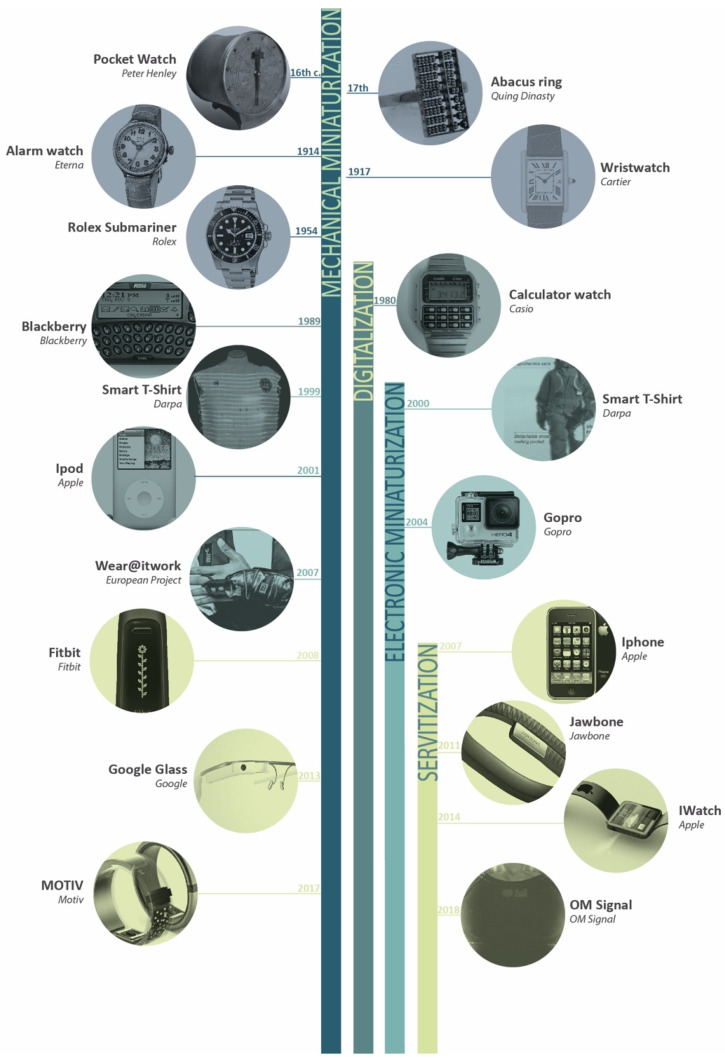
Wearables technological evolution along the history.

Figure 1 shows the evolution of wearables classified into the different technological phases: (1) mechanical miniaturization, (2) digitalization, (3) electronic miniaturization, and (4) servitization. As Figure 1 shows, wearables have been changing along the history both due to technical innovations and requirements as well as to specific human requirements. These technological innovations are mutually related stages that overlap over the time. That is to say, the mechanical miniaturization starts from the beginning of wearables history and it continues being a technological challenge nowadays. The same thing happens with the digitalization that started in the 1980s, with the electronic miniaturization that started at the beginning of the 21st century, and with the servitization that started almost 10 years ago. They all remain technological challenges nowadays. 

The beginning of the wearable era is marked by the appearance of several watches that were launched to the market improving functionalities of previous versions [4]. At first, the innovation was based on reducing the size of an existing product in order to make it smaller, and hence, to make it wearable. Then, new functionalities such as the alarm, the interval measuring ability, or waterproof features were added. Those innovations were developed in order to meet specific requirements set out by humans. Another example of an improvement in income was identified as a result of innovations during the following years such as the smallest production wristwatch with a “baguette” movement or an eight-day alarm watch [5]. Necessities of the 20th century were a factor in making wristwatches acceptable by the civil population and not only by soldiers. Thus, this first era was marked by the mechanical miniaturization of different mechanisms.

The advent of digitalization led to an improved performance of wristwatches and to the appearance of new wearables devices such as hearing aids [6]. By the end of 20th century, with the popularization of the use of internet, the portable phones functionalities were also improved and the email service was added to the phone firstly in the BlackBerry [7]. At this point, wearable devices were able to send and to receive data thanks to internet and to new telecommunication nets. Last 20 years have been marked by new technological developments that have led to create new challenges and that have been adapted to mass production. Electronic miniaturization, monitoring abilities, and the appearance of e-textiles have resulted in new wearable devices rushing to the market at a high speed [8].

Little by little, technological challenges have led to new human needs and those needs have driven the development of new technological solutions. Nowadays, the wearables are service hubs that can act both as active or passive full interfaces. For example, in the case of fitness trackers, as they provide information without the active engagement of the users, they are always providing information. However, wearables can also act as active interfaces, such as Google Glass that can be used for actively engaging with its service [9].

Finally, in the case of last smartphones or smartwatches, they act both as active and passive interfaces so they form complex networks of personal data that are carried by people.

This current situation has opened new research fields and challenges in the field of wearables and even more in the study of wearables acceptance or adoption in people. 

After having studied the evolution of wearables, it can be concluded that wearables’ design requires the analysis of different multidisciplinary methodologies to handle the issue of wearables throughout a user-centered approach. The following section describes the different methodologies that have been considered to develop the methodology presented in this paper.

### 2.2. Wearable Design Methodologies

This section presents design methodologies that can be applied in the development of wearables.

There are few studies that has focus on human-centered design for wearables development. In 2007, Knight proposed a methodology for assessing the effects of wearing a computer in terms of physiological energy expenditure, biomechanical effects, discomfort due to musculoskeletal loading and perceptions of well-being through comfort assessment [10]. Canina and Ferraro have addressed the importance of considering not only engineering aspects, but also the psycho-physical well-being of users [11]. Contreras-Vidal et al. have expressed the need to take a human-centered approach to improve interaction and to make wearables more effective, reliable, safe and engaging [12].

Accordingly, another similar focus aligned with user and body-centered research of wearables was developed by Bhömer in the doctoral paper “Designing Embodied Smart Textile Services” [13]. The research explores how close-to-the-body products and services can become meaningful to people. Along the line of embodied design, Tomico provided a discussion about the opportunities and challenges of designing soft wearables applying notions of personal-meaning–making through different design cases. Furthermore, in terms of design methods, Danielle Wilde et al. presented a framework that enables designers to understand embodied design ideation practices.

In complex products and hybrids design, the identification of design requirements gains importance due to the different factors that influence on their design. Some studies have developed solutions in software design [14] but wearables’ design stays unexplored.

Taking this challenge into account, this section includes theoretical principles from: (1) user-centered, (2) systems engineering, (3) industrial design, (4) interaction design, and (5) design innovation. These different methodologies can be merged into one, taking different issues from each of them.

In the framework of user-centered methodologies, different approaches have been explored: design thinking methodologies, co-design methodologies and embodied design methodologies. Additionally, general user-centered standards were explored [15].

Regarding design thinking category, the Double Diamond Model [16] has been taken as a referent for design stages definition. Due to the fact that it is a graphical model, the different convergent and divergent phases are easily differentiated. Additionally, different studies about design thinking interpretation and applications were explored [17].

From the participatory and co-design category, the Co-Create project funded by the European Union [18] has been taking as a referent to explore how the design methodology can be transferred to designers and practitioners. By exploring the co-design best practice report [19], key steps concerning team diversity and favorable environment creation were identified.

Regarding the embodied design category, some referent studies were considered [13,20,21,22,23,24,25]. This analysis helps to understand the importance of personalization and embodiment in wearable design. Moreover, most of them were indeed linked to wearables’ development.

In these studies, as it happens in user centered studies in general, the role of prototypes is pointed out. This fact has greater influence in the field of wearables since prototypes can be used not only to test their performance, but also to explore how users interact with them. Hence, prototypes constitute a core source of useful information for both design and validation process.

In the same way, computer science developed their own iterative methodologies where they experiment with users from the very early stages. In this field, prototypes are not always physical but digital, so iterations happen at a faster rate. Due to this reason, several useful definitions about verification and validation systems can be found [26].

Concerning industrial design methodologies, several references about the technical development and technical alternatives can be found. From engineering design methodologies, key development points were identified [27]. Included in this group there are also specific design methodologies for Internet of Things (IoT) development [28] and smart clothing development. From these methodologies, particularities from connected products and smart clothes can be obtained.

With regards to interaction design, service design and user experience methodologies were explored. Some of them are general service tools [29] while others are focused on IoT user experience [30].

As it can be deduced, it has been an integrative process.

Additionally, the theoretical framework construction is represented through a transit map that is represented in Table 1 and Table 2 present the methodologies description and the application to this paper. As it is shown, there are some common points between different methodologies. Above all the features represented, use of prototypes, iterative process, design/validation process and design methods, and tools/elements should be highlighted as common confluence points of different methodologies.

### 2.3. Wearable Design Toolkits

Once the methodologies have been explored, it is important to tackle the issue of how this methodology is communicated and transferred to researchers and practitioners. While in other fields such as organizational engineering gamification-based activities are commonly used as knowledge transfer tools, in design, toolkits, particularly card-based toolkits, are becoming increasingly common. The evidence and feedback from trials indicates and there are several advantages and that they engage participation [32,33]. The use of physical tools facilitates interaction and improves the usability of design methods. This section analyses different commercial design toolkits that can be considered referent models for design practices. Most of them are composed of different design methods that are represented in different cards. Only few of them include templates and guidelines about the usability of the tool. None of them includes thinking material, prototyping material and basic toolkit material. In addition to existing Wearable Toolkits, specific solutions addressed to the design of wearables have not been found. However, some references aimed at IoT idea generation have been identified.

An example of this, the IoT Toolkit by Postscapes [33] which is a digital platform that consisted of selected online resources to explore IoT products. As two relevant examples of physical toolkits, the IoT Tiles Cards and the IoT Service Kit should be mentioned. The IoT Tiles Cards [34], consist of a set of cards to engage users in ideation sessions for IoT product development. Similarly, the IoT Service Kit [35] provides useful information for creative sessions. Additionally, this toolkit includes 3D printed tokens that acts as sensors and other physical elements.

On the basis of the described methodologies and toolkits, the following section will be focused on the construction and integration of the theoretical background into a methodology to co-design and co-evaluate wearables.

## 3. Materials and Method

This section describes into detail the different studies that have been carried out to develop the methodology. Additionally, different users (researchers and practitioners; specialized experts and multidisciplinary experts, intermediate users and final users, etc.) have been involved through the different studies.

Outcomes from previous published studies [3] help to define the requirements of the co-design methodology. As mentioned before, the co-design methodology will be represented as a toolkit. Therefore, on the one hand, there will be requirements relating the methodology itself, and on the other hand, there will be requirements regarding the toolkit. The requirements are listed below.

### 3.1. Methodology and Toolkit Requirements

#### 3.1.1. Methodology Requirements

R1: The methodology should consider both human and technical factors during the design process.

R2: The methodology should combine verification and validation processes.

R3: The methodology should consider the whole user experience and enhance their involvement throughout the design, development and validation process.

R4: The methodology should integrate prototypes from the beginning of the design process.

R5: The methodology should combine divergent and convergent thinking techniques and quantitative and qualitative methods.

R6: The methodology should be a co-design process considering the participation of different experts from different fields.

R7: The methodology should be accessible for design and engineering practitioners.

#### 3.1.2. Toolkit Requirements

R1: The toolkit should be sequential and evolutionary.

R2: Although following an evolutionary process, it should be flexible enough to adapt to different contexts and experts’ profile.

R3: It should encourage dialogue between different players.

R4: It should include information about specific design tools.

R5: It should include templates to guide participants on the compliance of design techniques and clarify expected outputs of each step.

R6: It should include physical thinking material.

R7: It should be easy to transport.

### 3.2. Design Inspiration and Implementation Process

The theoretical framework shown above led to establish a design direction for the methodology and to analyze how different theoretical approaches can be integrated into the methodology. However, to meet both the methodology and toolkit requirements, it is necessary to gather spontaneously contributed ideas to innovate in the way the toolkit is presented.

Due to this reason, a brainstorm was made in order to gather spontaneous ideas. The reason of having chosen a brainstorm is due to the necessity of having multiple solution-based techniques that explore different options to transfer the methodology to users. As it has been seen in Figure 1 there is a high presence of game-related ideas in the brainstorming.

While card-based toolkits are broadly used in creativity sessions, game-based toolkits remain unexplored. Although there is widespread interest in the learning and motivation benefits of serious games (which means games intended to support learning), most of these applications have been studied mainly in pedagogical and educational fields [36].

Based on the characteristics and toolkit requirements above, the application of serious games in design methodology has special interest. On the one hand, this is due to their integrative role and, on the other hand, as generative methods of knowledge that help to enhance the co-design between different stakeholders.

As an example of this application in design, The Lego^®^ Serious Play^®^ can be noted. This method is a technique, which improves group problem solving. By utilizing visual, auditory, and kinesthetic skills, the Method requires participants to learn and listen, and it provides all participants with a voice [37]. This is a great example of the effectiveness in the use of serious games to create innovative solutions [38]. 

What we want to achieve is a fundamental serious game not only to enhance creativity but also to help the approaching of the design methodology to participants during all the design stages.

**Figure 2:**
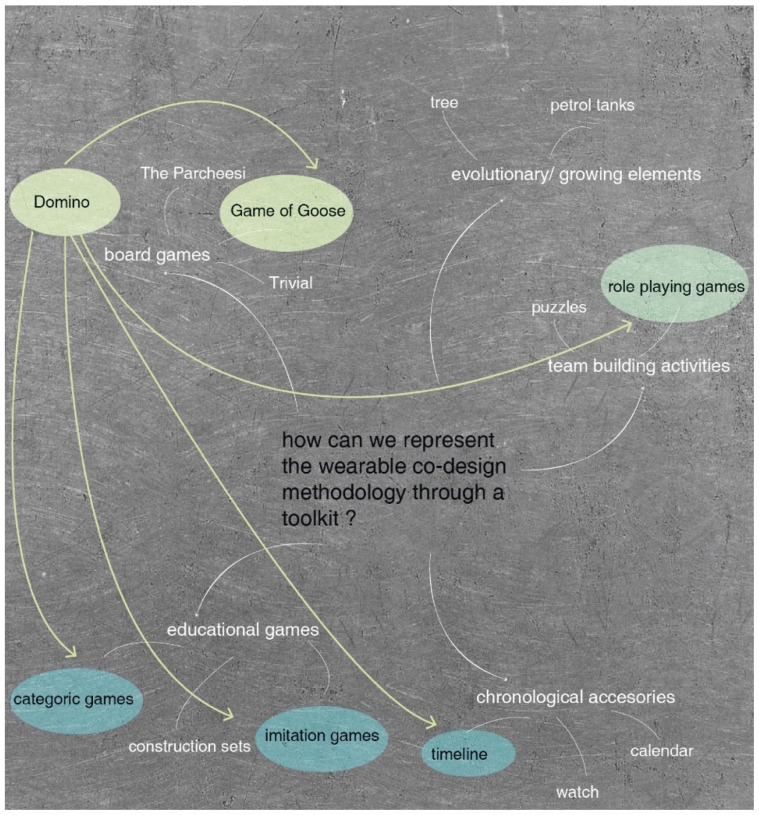
Results from the Brainstorming on the toolkit development.

In order to find a design solution considering all the different inputs of the previous analysis, a brains storming was made and it is represented in Figure 2.

As a result of the brainstorming, different games and educational elements were suggested. As it can be seen in. The Domino stands out from the brainstorming. As we know, domino cards are represented as a rectangular tile with line dividing its face into two square ends. Each end is marked with a number of spots or is blank. The logic behind this game, keeps several common points with the methodology procedure, which is to be established. 

We suggest using the domino cards as card-based toolkits where different design techniques are represented. Thus, in each domino cards different design techniques can be represented. The different numbers marked in both faces helps to follow the design process of wearables. In some cases, there are different domino cards which engage with the previous card. This will provide the toolkit with the flexibility to adapt and personalize the design process according with the context of application of each wearable.

Once the design direction is established, it should be landed in a methodology. To that end, following steps have been identified:

**i.**  Plan the wearable design process. Turning to the definition given in the Introduction, we can further differentiate two product types: a physical product and a digital product that can be defined as follows:
  (1) Physical product: The physical part of the wearable in contact with the user. Hardware and other holds are considered in the product. Example: the hardware of a smart glove.
  (2) Digital product (service): Common part where the user and the product interact. Each touchpoint of the gadget that allows coming to the service through communication or interaction. Example: the user experience of the smart glove.

Taken these definitions into account, the planning of the process is differentiated by the physical product and digital product development. The full process can be divided into six different stages: (1) ideation, (2) service development, (3) service test, (4) physical product development, (5) physical product test, and (6) delivery.

**ii.**  Ideate the task/function mapping. Combining the design concept and the planning of previous point, it can be determined that each phase is divided into different stages where different design tools are offered to players. These tools are shown in independent domino cards that players have to choose according to their preferences or specific requirements of the project. In each stage, there are templates available as well as references and toolkits. At the end of each phase, there is a verification point where users have to complete a checklist in order to assure that they are meeting ergonomic and design requirements. These points are iterative. If requirements are not fulfilled, players have to go back to previous stage. by the end of the game, players should be able to fill out a develop brief that will gather the main steps to develop a wearable.
**iii.**  Define the key iteration stages. The key iteration stages are defined by prototypes and by co-evaluation checkpoints. However, it should be considered that, in a real process, iterations can happen at random. The methodology is prepared to collect all the modifications resulting from each iteration in concept re-definition templates described in point 2.
**iv.**  Identify stakeholders. It is recommended to have a minimum of four players and a maximum of eight players in the “wearable design domino”. The minimum quantity of players is defined based on the different profiles that should be covered. The maximum quantity is defined to avoid overcrowded session. Additionally, it is important to have a homogeneous distribution of profiles. Each stage is led by a different player. The roles that should be covered are the following:
  (1) Designer (industrial designer or interaction/service designer)
  (2) Engineer (industrial engineer or informatics engineer or electronic engineer)
  (3) Ergonomist (physical or cognitive and emotional specialized ergonomist)
  (4) Stakeholders (intermediate users or final users)

The options show in brackets represent the different suggested recommendations in case of having eight players.

**v.**  Understand and specify the context of use. Although the methodology has been constructed based on an empirical case with industrial applications, further applications such as sport or health related fields could be explored. In these cases, the players roles might change (for instance, instead having the ergonomist a physiotherapist or a doctor could be joined).

## 4. Results: The Wearable Co-Design Domino

As a result of the design process, the wearable co-design domino is presented in this section.

The wearable design domino is a non-hierarchized methodology to co-design wearables with the active involvement of users and different experts. Domino pieces represent different design techniques that should be chosen in consensus among different players.

### 4.1. Formal Qualities

The formal qualities are the different components of the methodology. Each component is a physical element that integrates several theoretical concepts. The conceptual schema is shown in Table 3.

#### 4.1.1. Domino Cards

Domino cards are different pieces where design and ergonomic techniques and tools are represented. The techniques description is written in English. The content is based on the findings of a previous study realized by Frances et al. so the list of tools provided in previous paper is represented in the Wearable Co-design Domino as different domino cards. The top of each card is marked with a number of spots that represents the order of the process. Additionally, each phase is represented through a different color to facilitate the interaction with the tool. The back side includes a description of the techniques, outcomes a definition of players and leader and, finally, the available information is shown in the right corner. The leaders are defined according to the field of specialization of each player. The available material indicates the existence of three different items: templates, references, and prototyping toolkits.

The main goal of domino cards is to evoke actions and idea generations. The leader definitions makes the We Co-Do enhance the equal and inclusive use of the tool. As explained before, there are different domino cards which engage with the previous card. In this case, players have to discuss and decide which card are going to use. This means that the dialogue between different roles involved is encouraged constantly during the game.

**Figure 3:**
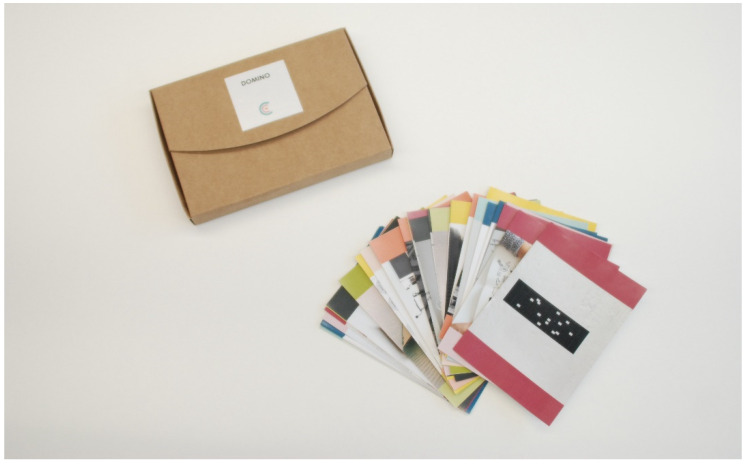
Photo of Domino cards.

**Figure 4:**
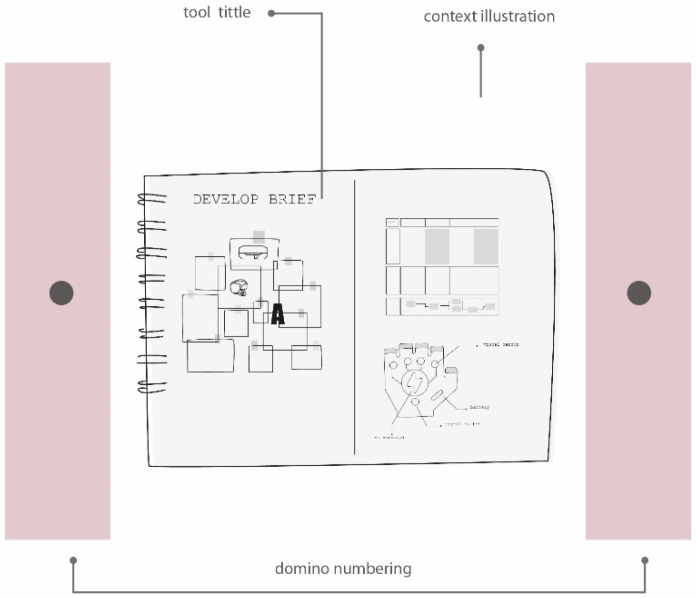

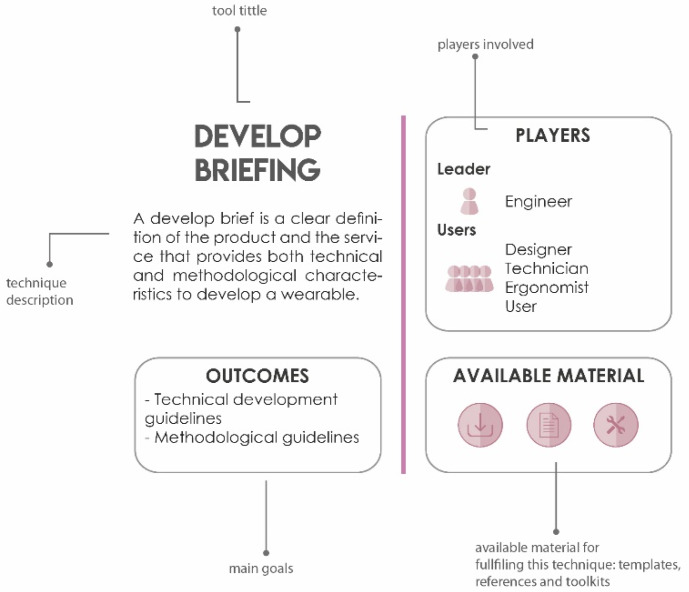
Content from the top and back side of the domino card.

Figure 3 shows the general view of domino cards and Figure 4 shows the content of each domino card. As can be seen, in the top size, the domino card title and a contextual illustration are presented. Alongside the illustration, the numbering of the domino card is also included. In the back size, the design or ergonomic technique description and the outcomes are described. Additionally, the different players role and the available information are included.

#### 4.1.2. Templates

Templates are provided in each stage. It consists of detailed files with different sections in order to complete the domino cards properly. In each section different tasks are asked to be completed. In some cases, additional information and terms definitions are given to participants. The tasks included evocative questions that help the participants to fill all the gaps.

The main goal of the templates is to collect all the information and design decisions during the design process. It is a way of having the traceability of the process. Each template is designed according to the goal of the domino cards. 

**Figure 5:**
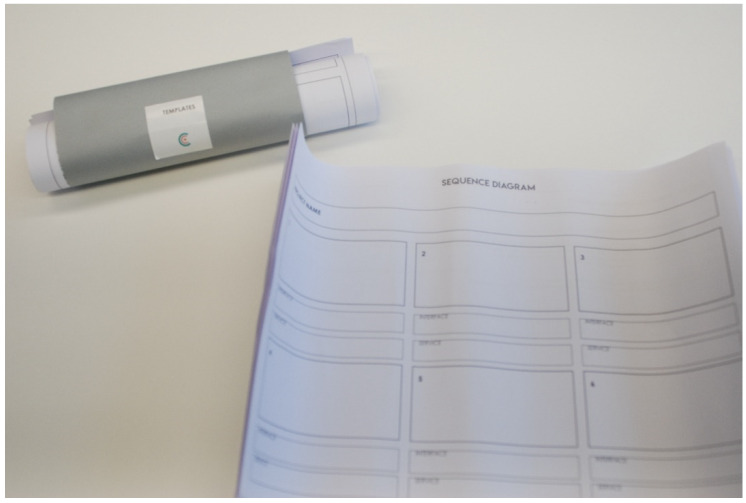
Service Diagram Template.

**Figure 6:**
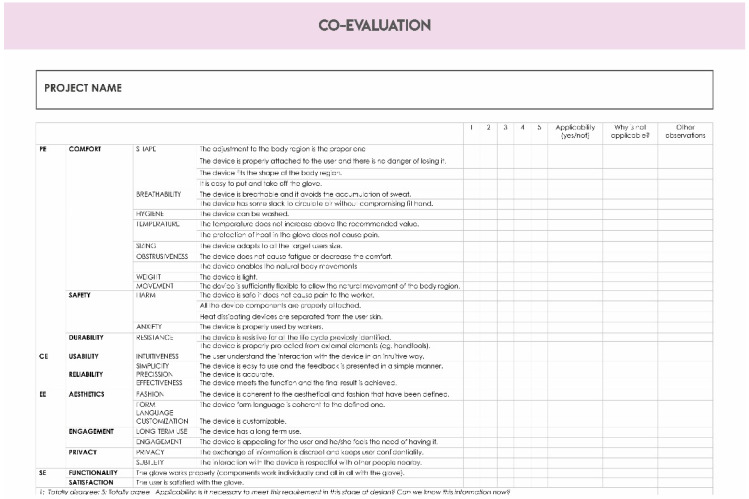
Content and structure of the Checkpoint template.

Figure 5 shows the photo of the service diagram template and Figure 6 shows the content of the checkpoint template.

#### 4.1.3. References

References provided additional information to complete each stage as well as different resources that can be selected by players according to their requirements. It constitutes assets of physical and digital resources for some of the domino cards. 

**Figure 7:**
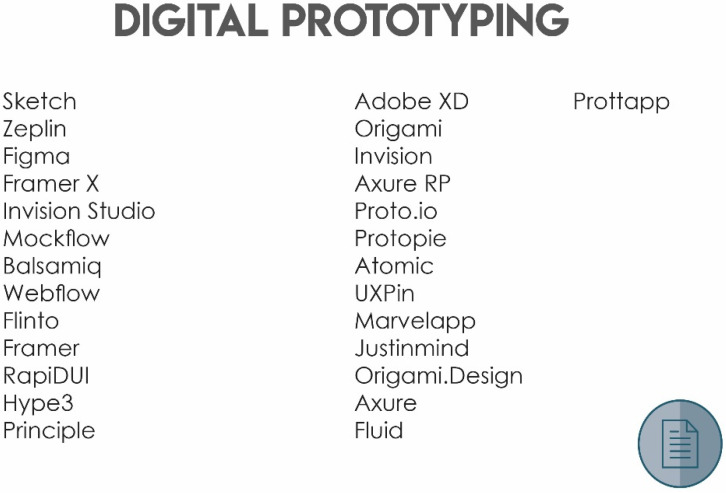
Example of a reference card.

Figure 7 shows the digital prototyping card.

#### 4.1.4. Prototyping Toolkits

Prototyping toolkits, include physical and digital prototyping resources as well as basic workshop material.

**Figure 8:**
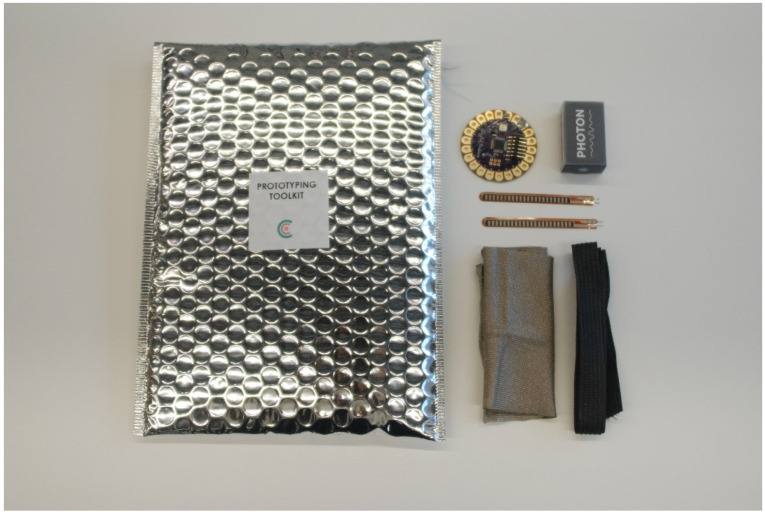
Prototyping Toolkit included in the Wearable Co-Design Domino.

The basic workshop material case includes some pens, post-its and basic items used during the We Co-Do. The prototyping toolkit includes functional prototyping material, such as some motherboards, conductive threads, e-textiles, and different sensors. Figure 8 shows a photo of the prototyping toolkit.

#### 4.1.5. Handbook

**Figure 9:**
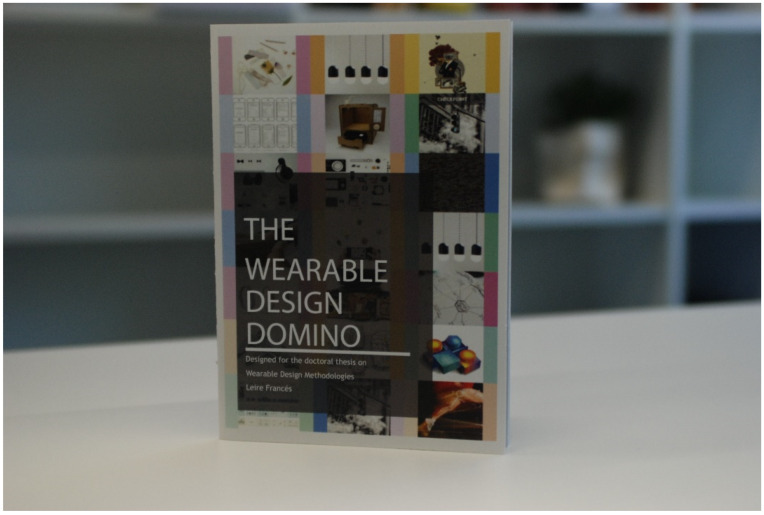
Photo of the We Co-Do Handbook.

The handbook presents the guidelines for completing the Wearable Co- Design Domino. It includes methodology description, formal qualities as well as players’ rules. Through the methodology description, we want to enhance the co-design of players. The formal qualities describe the content of the wearable co-design domino and, finally, the rules are some basic guidelines that should be kept for the proper workability of the wearable co-design domino. Across the handbook, a brief summary of this paper is explained. Figure 9 shows the photo of the handbook included in the wearable co-design domino.

### 4.2. Wearable Co-Design Domino Layout 

**Figure 10:**
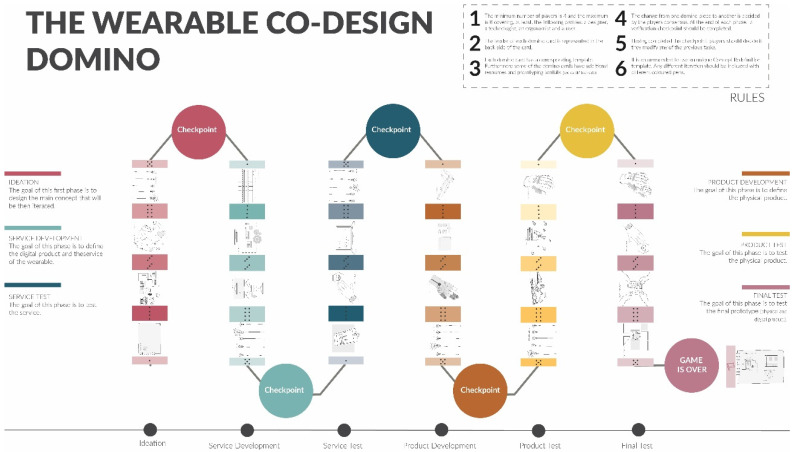
General view of The Wearable Co-Design Domino.

As it has been described above, one of the main components of The Wearable Co-Design Domino are Domino Cards. Figure 10 shows the example of an ended process. The six different stages of the design process are represented, each of them in a different color code. As described in Section 3.2. Design Inspiration and Implementation Process, the different stages are:

- Ideation
- Service development
- Service test
- Physical Product development
- Physical Product test
- Delivery

In each phase, four different cards have been included. At the end of each stage, there is a Checkpoint where participants have to complete the Checkpoint template. 

**Figure 11:**
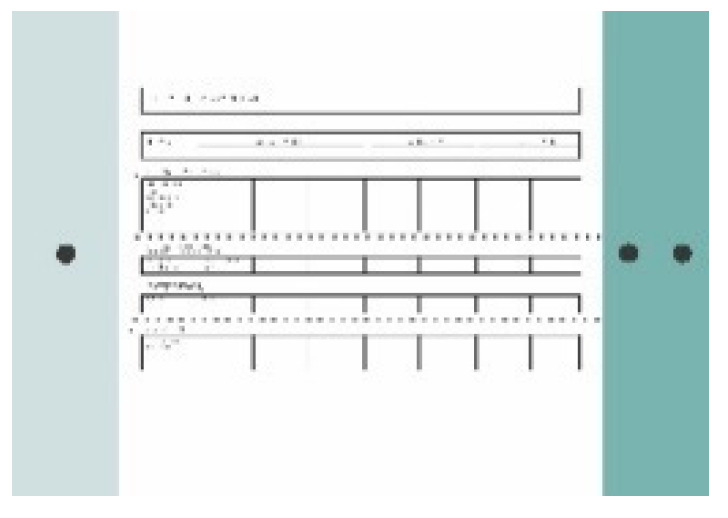

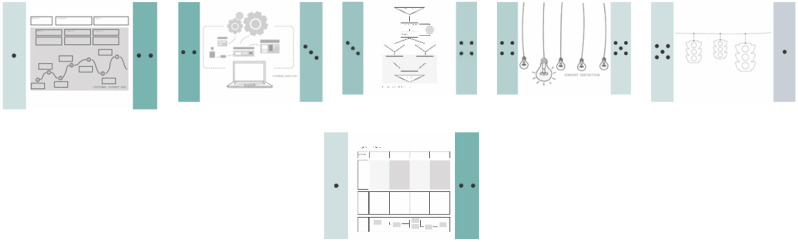
Example of Service Development Domino Cards.

As explained in Section 3.2, the methodology follows the dynamics of the domino play. That means that in each step there are more than one technique (or domino cards) that can be applied. Figure 11 shows the example of the service development stage. As it can be seen in the figure, in the first step there are three different domino cards (service blueprint, interaction map and customer journey canvas) that can be used. Participants, should select one or more than one domino card according to the project or their experience.

## 5. Validation

In order to test the methodology, we engaged multidisciplinary volunteers and design practitioners in a co-design workshop and asked them to develop a smart glove. The identification of the design experts was made in collaboration with EIDE (Basque design association) [39], who suggested the design experts that were involved in the wearables field. The design brief for the workshop was based on the real empirical case carried out in a previous study by the author consisting of a “design and development of a low-cost smart glove to track forces exerted by workers in an car assembly line” [40].

We made a first pilot workshop with internal volunteers and a second workshop with design and engineering practitioners. Each workshop test different hypothesis that are described into detail below. User workshop as a cooperative evaluation of the methodology. The chosen method was a user performance trial which was carried out by applying different methods to collect and observe participant responses [41].

### 5.1. Internal-Workshop

The main goal of the pilot workshop was to test the timings; domino cards templates and prototyping material utility, and general usability of the tool. As time was compressed and the workshop lasts 3 h, a pre-selection of the material was made by the researchers. Although the methodology consisted of allowing the election of domino cards in hands of players, this step was made by researchers in order to focus on the defined objectives of this pilot workshop. A total of 10 domino cards and templates were chosen to this workshop. 

Thus, based on the describes goals, the hypothesis to test in this workshop were:

- Definition of the required timings to complete the different tasks.
- Usability of domino cards, templates and prototyping material.
- Necessity of creating additional supporting material.
- Testing the comprehension of the different terms and tasks.

### 5.2. Participants

A total of six participants were involved in the pilot workshop: three design researchers (a facilitator and two observers) and three engineers (internal volunteers who has to face the challenge).

The role of the facilitator was to guide the workshops, to manage the workshop timings and to help the volunteers in the accomplishment of the tasks. The two observers were taking notes about the performance of the workshop, interaction between volunteers and the tool, and the volunteers’ general perceptions.

### 5.3. Development

The chosen method was a user performance trial. The three participants were given a design brief to design a smart glove. 

The chosen domino cards and templates were: brainstorming, concept definition, sequence diagram, software architecture, anthropometric study, components selection, prototypes making, and three co-evaluation checkpoints.

**Figure 12:**
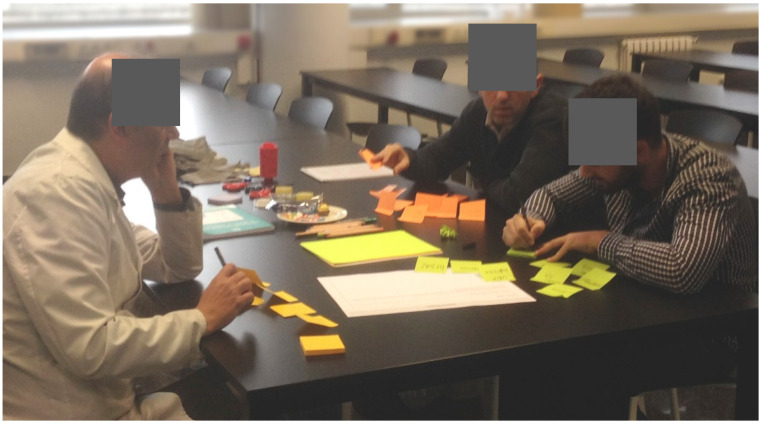
Internal workshop participants during the Brainstorming.

**Figure 13:**
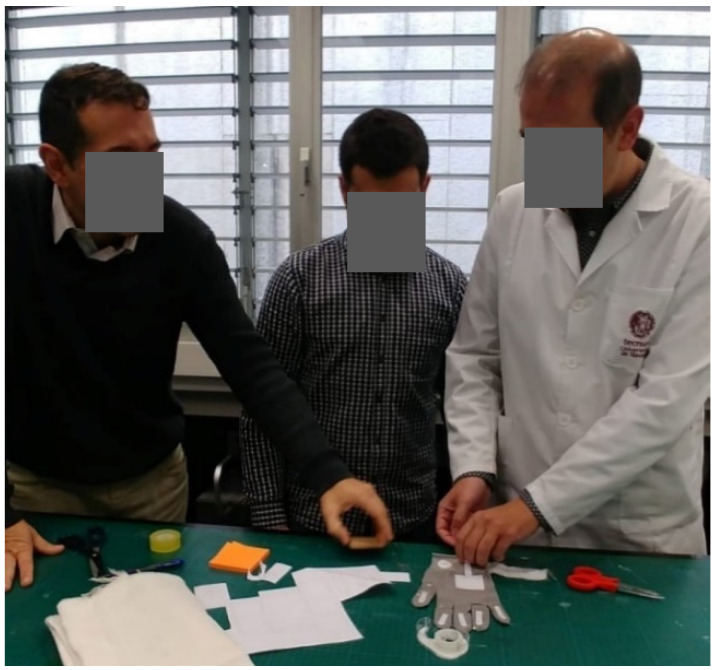
Internal Workshop participants during the Low-Fidelity Prototype Development.

**Figure 14:**
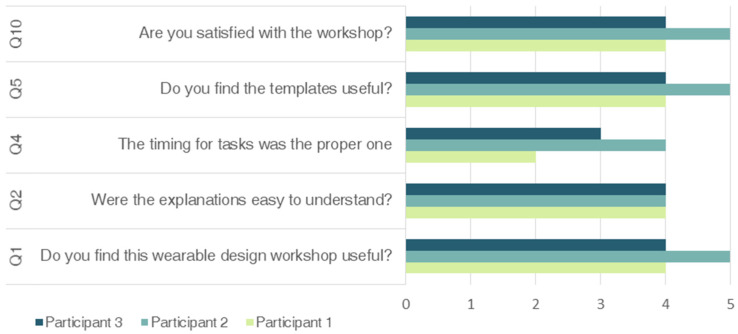
Participants’ answers on categorical scaled questions.

Each domino card and template have a scheduled timing. First of all, 5 min were given to volunteers to read the domino cards and then, around 20–40 min to complete the template. All the doubts that cropped up during the workshops were clarifies by the facilitator while the observers note them down. Figure 12, Figure 13 and Figure 14 show the participants realizing the prototypes during the elaboration of prototypes.

### 5.4. Data Collection Method

How the data is collected is almost as important as the how the workshop is developed. There are few studies focusing on research triangulation of design processes, despite its fundamental role in theory building/testing cycles [42]. In the case of this workshop, triangulation was applied by using different data collection methods to compare and contrast result in different way. Thus, written diaries were used by observers, then feedback canvas templates were completed both by observers and by participants and finally, online surveys were sent to participants. Formulated questions are shown in Table 4.

### 5.5. Results

Regarding the observers’ diaries, main insights were regarding the timing of the tasks and the facilitator’s explanations. Timings resulted very tight and the facilitator had to explain most of the templates and design techniques collected in the domino cards. They also had few spaced in the templates for writing everything. Although the order of the domino cards and templates were predefined, they changed the order of same templates in certain points: for example, the concept definition, and sequence diagram templates were used two and three times consecutively. This happened due to be an iterative process where the concept changed several times throughout the development of different aspects. Additionally, when participants were using the co-evaluation checklist, they also changed the concept and, therefore, the concept definition and sequence diagram templates.

As a conclusion, it can be said that some of the templates are static (do not change with the concept change) and other dynamic (they change with the concept change). Finally, although the roles were not defined, they naturally adopted different roles. One the volunteers adopted the role of the ergonomist (he was all the time thinking about the usability of the smart glove in the working station), other one adopted the role of the technical expert (he was thinking about the performance and viability of the smart glove) and finally the third one (who was a design engineer) adopted the role of designer. The facilitator guided and explained some of the techniques and contribute to new creative ideas. The team involvement in the task was, in general, very satisfactory and allowed the observers to collect very useful information.

Regarding the feedback canvas, the results were aligned with the observers written diaries. Generally, they valued positively the fact of co-designing and team working. Otherwise, they found difficult to complete the tasks on time and to fulfil some templates without having assumed previous steps. Some doubts were generated regarding some of the terms used in the templates. As a suggested idea, they proposed additional clustering and summarizing steps. 

Finally, online surveys provided detailed information about the insights gathered. Figure 10 and Table 5 summarizes the survey results; firstly, the question with rating scales are shown in Figure 10 and secondly, questions with words cloud format are shown in Table 5.

From the survey results, it can be concluded that both domino cards and templates, that is to say, those elements that constitutes the content of the methodology, were useful to fulfil the tasks. Anyway, as can be seen in Table 5, some improvements regarding some terms definitions and additional examples could be added. Moreover, since templates are linked to specific tasks, participants suggested to add more generic templates to make them think and summarize previous steps.

Regarding the timings for the templates, participants found it difficult to complete the tasks on time. Timing was so tight and the leap from one step to another resulted so fast. Considering that these workshops require compressing the time these insights is not considered determinant. In a real situation, the scheduling and timing should be adapted to the particular context of use.

In general, results were satisfactory since participants were completely involved in the task and were independent almost all the time. Figure 14 shows the participants’ answers on categorical scaled questions.

As a main insight of the data collections methods, it can be concluded that the fact of using three different methods help researcher to get contrasted results and to gain validity on insights.

The main goal of the expert-workshop was to test the usability of the complete tool. Suggested improvements of the internal-workshops were implemented. In this workshop, all the domino cards, templates and references were included in the workshops. The workshop was separated in two sessions lasting 3 h each. The design brief was adapted from the empirical case studied in this paper in the Study 2.

### 5.6. Suggested Improvements

Based on the results obtained during the workshop, improvements can be classified into: content-related improvements, usability-related and workshop performance related improvements:

Content:

- Including a glossary of terms. As the casuistry of participants’ profile may be very diverse, some of the terms can be unknown, and thus, misinterpreted. It could be supportive to include a terms’ glossary card or include definitions and examples in the different templates.
- Including a handbook. Although the facilitator of the workshop explains the general performance issues, it would be interesting to design a handbook including the content, structure and rules of the tool.
- Including additional generic templates. Although some of the templates such as concept re-definition or a sequence diagram are dynamic in the process, they are associated to specific domino cards, so participants use them when it is defined in the domino cards. Since it is an iterative process where ideas are developed and redefined a number of times, it would be interesting to define some dynamic templates as main templates.

Usability:

- Increasing the size of specific templates. In connecting with the previous suggested improvement in point3, it could be interesting to provide additional writing space in some generic templates, or to define double templates. Such templates may be visually appealing to be useful at all.

Workshop performance:

- Providing more time to complete the templates. This is a matter for discussion since the tool is thought to have a quick and general view of the wearable development through a co-design methodology. Otherwise, so tight schedules may press participants.
- Providing break time between different tasks. This is related to the need of having a general view of the process that is expressed by participants. Breaking times always help to put the tasks in context, and improve the incubation stage of the creative process.

### 5.7. Expert Workshop

While the main goal of internal workshop was to test timings and usability of the tool, in the expert workshop, the goal was to test the complete methodology and to gain insights from the content by experts with experience in different fields related to wearables. Thus, the aims were:

- To test the content and the usability of the full methodology.
- To evaluate the co-design and co-evaluation of the methodology by experts.
- To evaluate the performance of the workshop and to compare and contrast the results of two group of experts.

### 5.8. Participants

A total of twelve participants were involved in the expert-workshop: four design researchers (a facilitator and three observers), two ergonomists, two designers, two engineers and two users.

For this workshop, two groups were made. Each of the groups were composed of the main four expert profiles. Thus, both groups were composed by an ergonomist, an engineer, a designer, and a user and they had to complete same tasks simultaneously.

### 5.9. Development

The expert-workshop development was similar to the internal-workshop, but it was extended in time and include additional tasks and templates. A total of 15 domino cards and templates were given to participants. The chosen method was a User performance trial. The participants were given a Design Brief to design a Smart Glove.

Additionally, as the Smart Glove is targeted to a real assembly line, part of the installation (the rear gate of the car) was mounted on the Workshop space. The aim was to help participants not only with the physical prototyping but with the experience prototyping.

**Figure 15:**
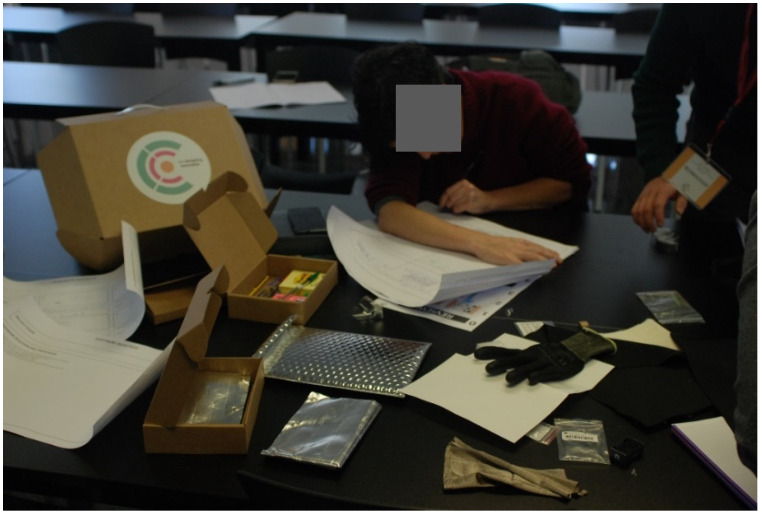
Participants from Group 1 completing the templates.

**Figure 16:**
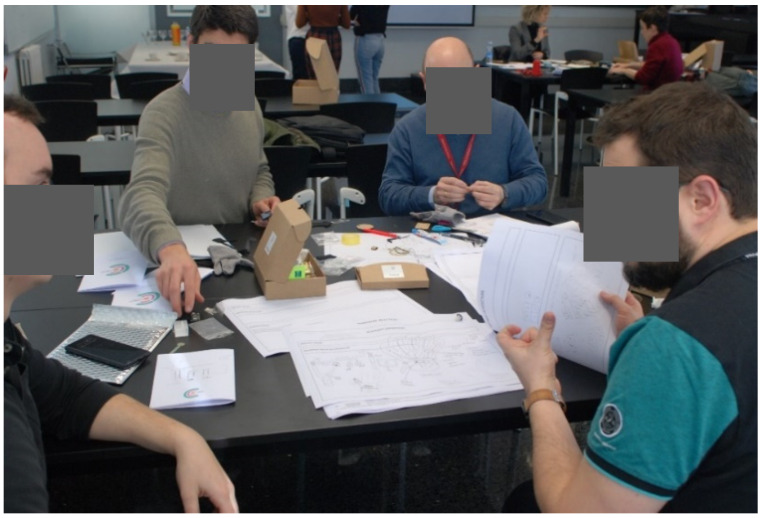
Participants from Group 2 completing the templates.

**Figure 17:**
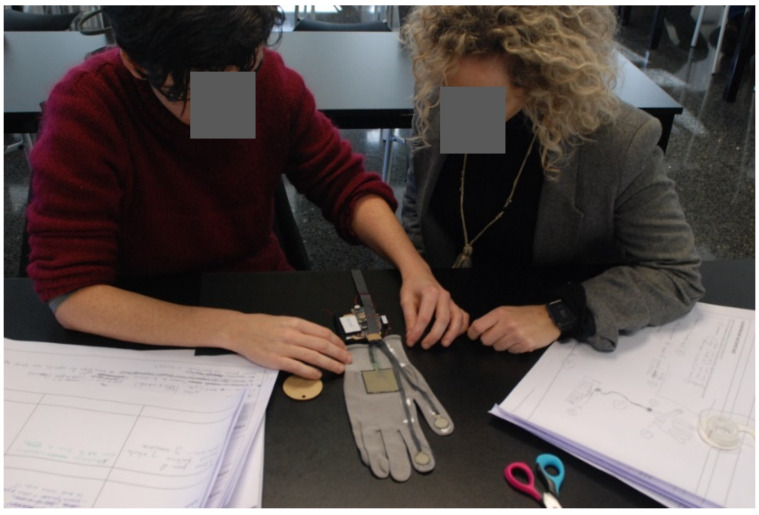
Participants from Group 1 making the physical prototype.

**Figure 18:**
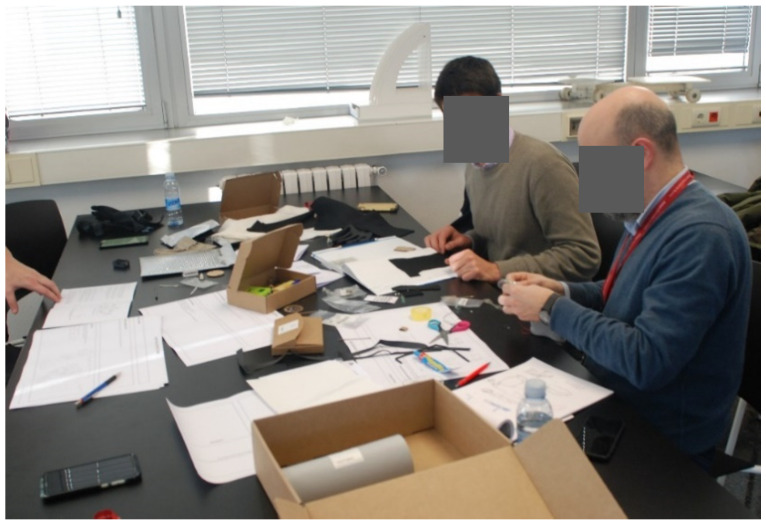
Participants from Group 2 making the physical prototype.

**Figure 19:**
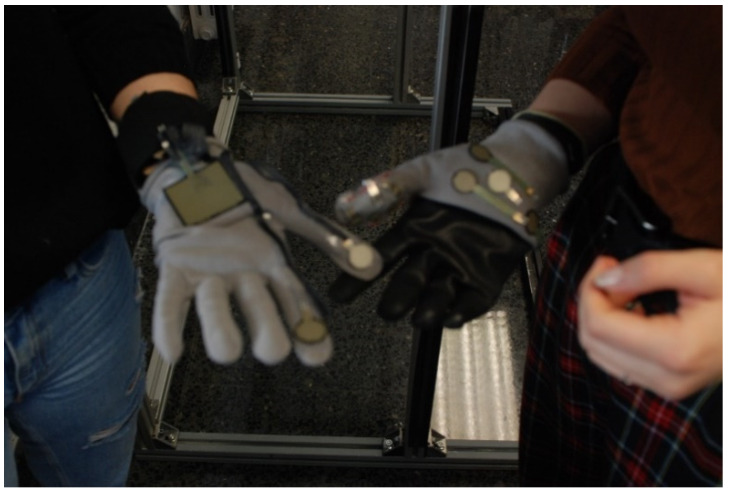
Left: Low-fidelity prototype developed by Group 1 Right: Low -fidelity prototype developed by Group 2.

The facilitator clarified all the doubts that cropped up during the workshops while the observers note them down. Some photos from the experts’ workshops are shown below. Figure 15, Figure 16, Figure 17, Figure 18 and Figure 19 show the participants during different tasks of the workshop.

### 5.10. Data Collection Method

For this workshop, individual structured diaries were prepared for both the observers and the active participants. The diaries included a feedback canvas divided into four different sections positive inputs, negative inputs, raised doubts and suggested ideas. Additionally, quantitative and qualitative questionnaires were included. In the case of the diaries for observers, a customer journey map was also included in order to collect participants’ interactions.

Regarding the qualitative information collected in observers’ diaries, it could be said that the time scheduling resulted tight still and that participants found it difficult to complete the tasks on time. Most of the comments gathered in the feedback canvas were regarding the definition of some of the players involved. Specially, the role of the “user” was uncertain and the participants with this role did not know what their positioning was. 

From the customer journey map, the perceptions were positive in general. In some specific moments, such as in the design brief, brainstorming, and co-evaluation checkpoint, observers wrote down that there where misunderstanding with the terms.

The analysis of the templates resulted very satisfactory. The information was written down in the specified space and almost all the templates were completed at all.

Regarding the participants structured diaries, it could be said that the workshop perception change depending on the expert profile. In general, designers and ergonomist find their selves comfort and easy to complete the tasks.

**Figure 20:**
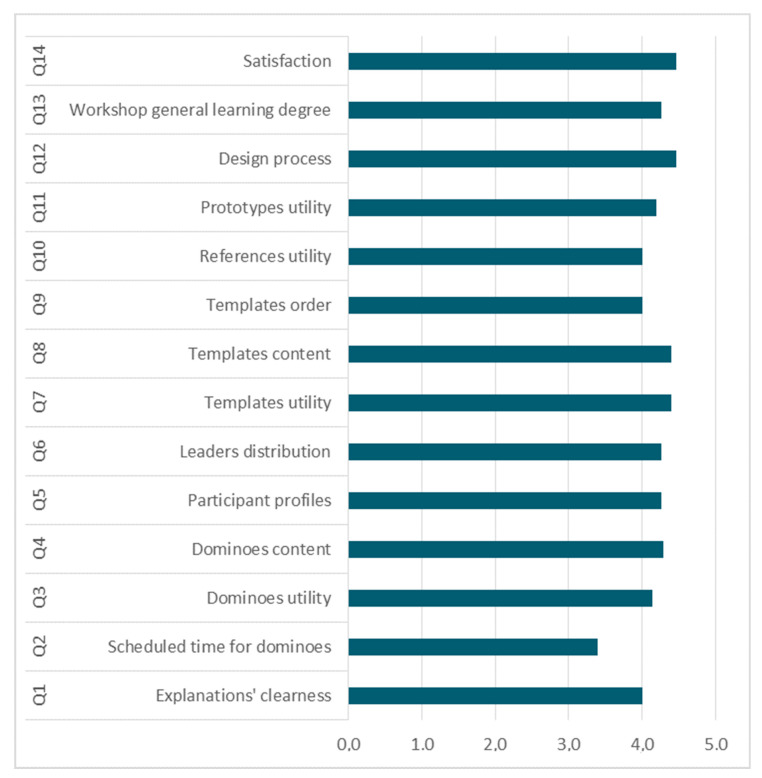
Participants’ answers on categorical scaled questions.

As far as the feedback canvas is concerned, they were satisfied with the workshop dynamic and the fact of having a roadmap to guide them in the design process. In word of some of the experts “the wearable co-design domino is a catalyst”. Most of the positive comments were concerning the process dynamics different enrichment on having different experts’ view and the intuitive and appealing design of the toolkit. As negative and improvement points, they highlighted the need of clarifying work dynamics of some design tools, such as brainstorming (importance of including the brainstorming key rules). Additionally, the visibility of references and definition of the design brief. Figure 20 shows the participants’ answers on categorical scaled questions.

## 6. Discussion

This paper offers a number of findings, and thus makes several theoretical and empirical contributions. This paper extends the application of human-centred design to the convergence of multiple approach methods by developing a methodology based on co-design and co-evaluation. This paradigm shed light on wearables device adoption both by final users as well as by stakeholders.

As it has been shown before, the first two papers support the object of study of this paper: the lack of dialogue between human and technical factors. 

Based on such challenges, in a previous study [40], we adapted an existing design methodology, The Double Diamond Model to the development of a smart glove. The contribution of the empirical case offers a twofold interpretation. On the one hand, from the product- oriented view, a physical contribution was made: a Smart Glove that could be used in real assembly lines. On the other hand, from a process-oriented view, a design methodology was established to develop the smart glove. This fact bridges the domain of product and process and, in parallel, the human and technical domains. It also breaks with the mistaken belief that technologically driven fields cannot be explored through user-centered approach. Otherwise, it helps to visualize how design methodologies enhance the productivity of processes.

Through the identification of wearable design requirements made in a previous study [3], the dialogue between human and technical factors was landed by suggesting the wearable design requirements wheel model. Thus, design requirements were categorized by physical, cognitive and emotional ergonomics rather than by technical or human factors. This classification helps to see each requirement as a combination of both factors and not as independent specialisms. Additionally, a design requirements approach was suggested to evaluate such requirements. This contribution opens a new direction of the design process since, from that moment on, the wearables design process was conceived not only as a design process, but also as an evaluation process. 

In this paper, every independent contribution gained meaning into a comprehensive methodology. The final and main contribution of this paper, consisted of a wearable co-designing and co-evaluating methodology that integrated the different outcomes obtained in previous papers. Insights from the double diamond model were used to plan and order the process, the wearable design requirements wheel model was used to create the co evaluation checklist and the evaluation approach was implemented by introducing prototypes and several verification and validation points in the process. It is a methodology based on the definition of a co-designing process that enables the existing ecosystem of human factors and technological factors.

Additionally, the way the methodology was developed, offered a value in terms of the methodology accessibility since it was represented as a toolkit. This fact makes the methodology more usable, and therefore increase the methodology acceptance between different experts.

### Implications

Based on the conclusions and contributions pointed out above, this section represents the implications for design researches, design practitioners and other fields.

Design research has to deal with many scientific challenges such as the increase of theoretical and methodological rigour [42]. In this way, beyond the final contribution of this paper, prevails the methodology that has guided this paper. The research through design process helps to understand the integrative role of design and, particularly, to bridge the academics´ and researchers´ points of view.

On one side, methods and tools developed by academics are required, and on the other side, validation by real practitioners is also needed. Accordingly, this paper has combined systematic literature reviews, theory-driven studies, real empirical cases, methodologies used for both design researchers and practitioners, and finally, several verification and validation procedures. This is particularly important for understanding how both qualitative and quantitative methods can be useful for addressing research questions [42].

The triangulation of methodologies used for the development of this paper allow researcher to connect diverse research methods and to have a referent model on formalized and rigorous design research.

Additionally, the co-design and co-evaluation approach enhance the coexistence of different fields in design projects and encourages dialogue between different experts. In this way, the wearable co-design domino could be used to foster the communication between different departments at the university or research and innovation centres. Furthermore, regarding professor–student relationships, the wearable co-design domino can be also used for as a knowledge eliciting tool whereby both co-designing values can be transferred.

Design entrepreneurs and practitioners have to deal with several daily living aspects that require high efficiency in their design process. Scheduling the project and creating budget is a costly activity that require deep knowledge about all the phases. In this way, the wearable co-design domino implies some advantages such as resources for scheduling a project, identifying possible partners and collaborations, and identifying technical and organizational constrains.

At another level, the wearable co-design domino could also be used in team-building activities to encourage dialogue between different experts, and thus to create participatory design culture. Additionally, for breaking barriers associated to the lack of design culture in associations, the wearable co-design domino could be used to fostering design practices in different types of entities.

## 7. Conclusions

The methodology is a tacit representation of several values to be implemented such as the equal and inclusive participation of different players, as well as different technical and design techniques.

This paper summarizes the main conclusions of the research work developed during this paper. We describe how the research questions have been answered and the contributions delivered in each study and paper. Later, the implications of such contributions are analyzed from the academics’ and practitioners’ points of view. Finally, the future research lines derived from this paper are presented.

In Study 1, theoretical literature evidence to the situation explained above was given. From the evolution of wearables along the history, it can be concluded that the development of new wearables devices is the result of a close relationship between technical and human requirements. Clearly, this conclusion can be transferred to the current status of wearables. From the analysis of current challenges, it can be concluded that the user acceptance of wearables depends on transversal relations. Although we differentiated technical challenges from human challenges, the final usability and satisfaction depends on a combined focus of both. Based on these insights, the main objective of this paper was to create a dialogue between human and technical factors during the co-design process of wearables.

To complement the theoretical literature evidence, an empirical case study was carried out. The design process of wearables requires a holistic view of all the factors involved. An existing human-centered model, the double diamond model was used to face this challenge. The adoption of this model helped to empirically contrast the identified insight from Study 1 and led us to explore how technological-driven fields can be explored through a user-centered approach. From the application of the double diamond model, there are several advantages. Firstly, the importance of identifying objectives and boundaries from users and stakeholders’ meetings and how these meeting helps to frame the technical literature review. Secondly, material selection is a body-centered issue, and thus the material selection follows physical ergonomics. Related to this, it also shows how the components placement has to consider human body shape and comfort issues and how different fidelity prototypes can give relevant information about the insights not only about the performance, but also regarding the interaction of prototypes and users.

To create a validation process, a methodology is required, and this is why this paper had an integrative role. How can we put in the process? How would we integrate? While in previous studies have been carried out dealing with the main challenge, the leading research question of the final study was, how can we co-design and co-evaluate wearables through a user centered approach? The final conclusion that the design process also requires validation and verification to happen simultaneously. This helps to understand the following:

- How design methodologies help to provide evidence rather than opinions (providing the value and relevance of methods).
- How multiple evaluation loops during development can avoid short and long-term failures and improve the cost–benefit ratio.

Taking those final conclusions into account, this paper contributes to provide a useful roadmap to wearable designers through a user-centered approach in order to improve and increase the user-acceptance of wearables.

## Figures and Tables

**Table 1:** Methodologies that summarizes the underlying elements integrated in this paper.

Methodologies	Description	Integrated Elements in This Paper
**User-centered design (UCD)** (Design thinking, participatory design and co-design, embodied design ergonomics)	It is an iterative design process in which designers focus on the users and their needs in each phase of the design process [31].	-Double Diamond Model. -Design Thinking principles -Convergent and divergent thinking stages. -Iterative process. -Design/evaluation simultaneously -Participatory and Co-design principles. -Stakeholders definition. -Use of prototypes. -Design methods and tools.
**Systems engineering**	It is a multi-disciplinary and integrative field based on systems principles and concepts that integrates scientific, information and management methods.	-Iterative process. -Design/evaluation simultaneously. -Use of prototypes. -System validation principles. -Multiple-variants charts examples. -Systems development processes.
**Industrial Design** (Technological Design, IoT Design, Smart and functional Products Design)	It is a projective field composed of several disciplines that synthetizes knowledge, methods and techniques to enable the mass production of a specific product.	-Development phases. -Technical key stages of development. -Use of prototypes -Technical validation principles. -Particularities of wearables and IoT products.
**Interaction Design**	It is the design of the interaction between users and products. The goal of interaction design is to create products that enable the user to achieve their objective(s) in the best way possible [31].	-Existing Human Computer Interaction methodologies. -User experience validation principles. -Use of prototypes. -Interaction methods and tools -Particularities of wearables and IoT products.

**Table 2:** Overview of existing design toolkits.

Tittle	Company	Content
IDEO Method Cards	IDEO	Cards
Amsterdam MediaLab Cards	Amsterdam MediaLab	Cards
Dinngo cards	Dinngo	Cards
Collective Action Kit	Frog Design	Cards, templates
Frogthink workbook	Frog Design	Cards
Manual Thinking Kit	Luki Huber	Foldable maps, labels, pens, patches, box, cardboards
What is Affordable Housing?	Domus	Felt chart, felt pieces, guidebook.
Trip Kit	Olivia Paden *(Art Center College of Design)*	“Physical products” Guides Step by step process
Let’s Flow	Rapsodia	Physical cards

**Table 3:** Conceptual schema of physical elements and theoretical concepts integrated in the methodology.

Methodology Element	Element Description
**Domino cards**	There are physical cards with design and ergonomic tools description and leader and players definition.
**Templates**	There are physical paper templates to support players on the domino cards accomplishment. There are key explanations about the different tasks.
**References**	References are given through physical cards that are associated to different domino cards.
**Prototypes Toolkits**	-Basic Workshop material -Low-fidelity prototyping material -High fidelity prototyping material
**Checkpoint**	The checkpoint is a specific template that should be filled out between each phase. It consists of a list of wearable design requirements and the design team should check the compliance of such design requirements during the process.
**Players**	They are the multidisciplinary experts playing different roles.

**Table 4:** Questions formulated in the online survey.

Questions	Rating Scale
Q1 Do you find this wearable design workshop useful?	Categorical scale *Nothing useful-Extremely useful*
Q2 Were the explanations easy to understand?	Categorical scale *Not all easy–Extremely easy*
Q3 Could you suggest improvements for the explanations?	Words cloud
Q4 The timing for tasks was the proper one	Categorical scale *Nothing defined-Extremely well defined*
Q5 Do you find the templates useful?	Categorical scale *Nothing useful-Extremely useful*
Q6 Could you suggest improvements for the templates?	Words cloud
Q7 Would you prefer to working without templates?	Words cloud
Q8 What do you think about the templates scheduling and order?	Words cloud
Q9 What do you think about the prototyping material?	Words cloud
Q10 Are you satisfied with the workshop?	Categorical scale Nothing satisfied-Extremely satisfied

**Table 5:** Participants answers to open questions.

Questions	Participants Answers
Q3 Could you suggest improvements for the explanations?	-Including examples in the templates -A general explanation and steps of the workshops before starting.
Q6 Could you suggest improvements for the templates?	-Regarding the software architecture template, further information about software and hardware definition.
Q7 Would you prefer to work without templates?	-No, I would be not able to suggest coherent ideas neither to identify problems nor find solutions. -No, they put you on context
Q8 What do you think about the templates scheduling and order?	-Well, maybe there could be additional generic templates to summarize the previous steps.
Q9 What do you think about the prototyping material?	-Very useful not only to prototype but also to think. -I found them a good tool to think fast.
